# Lactate-Induced ZMYM2 K529 Lactylation Stabilizes ZMYM2 and Promotes Platinum Resistance in Ovarian Cancer

**DOI:** 10.3390/ijms27114707

**Published:** 2026-05-23

**Authors:** Zhenlong Yuan, Lu Deng, Yuting Zhao, Enyu Tang, Baofang Zhang, Shengnan Wang, Ning Li, Jing Yu, Lingying Wu

**Affiliations:** 1 Department of Gynecology Oncology, National Cancer Center/National Clinical Research Center for Cancer/Cancer Hospital, Chinese Academy of Medical Sciences and Peking Union Medical College, Beijing 100021, China; yzlbetablocker@gmail.com (Z.Y.); 18217721131@163.com (L.D.); b2023003100@pumc.edu.cn (Y.Z.); b2023003101@pumc.edu.cn (E.T.); zbfzzubetter@163.com (B.Z.); 13840598176@163.com (S.W.); lining1502@csco.ac.cn (N.L.); 2 Department of Clinical Laboratory, National Cancer Center/National Clinical Research Center for Cancer/Cancer Hospital, Chinese Academy of Medical Sciences and Peking Union Medical College, Beijing 100021, China

**Keywords:** ovarian cancer, platinum resistance, lactate, lactylation, ZMYM2

## Abstract

Platinum resistance remains a major obstacle in ovarian cancer, yet whether abnormal glycolysis and lactate metabolism drive this phenotype through protein lactylation remains unclear. Here, we investigated the role of lactate-driven protein lactylation in platinum resistance and sought to identify the key effector event involved. Global protein lactylation was assessed by immunohistochemistry in tumor samples from 122 patients with high-grade serous ovarian cancer, and integrated proteomic and lactylomic analyses were performed in fresh frozen tumors from 12 patients, followed by validation in ovarian cancer cell models and functional assays. Platinum resistant ovarian cancer exhibited enhanced glycolysis, increased lactate accumulation, and elevated global protein lactylation, which was associated with platinum resistance and shorter progression free survival. Integrated lactylome profiling identified ZMYM2 K529 lactylation as a platinum resistance associated event, and ZMYM2 was upregulated in platinum resistant tissues and cells. Mechanistically, lactate promoted ZMYM2 K529 lactylation, suppressed ubiquitin–proteasome mediated degradation, and increased ZMYM2 stability and abundance. Functionally, ZMYM2 enhanced cisplatin tolerance, homologous recombination repair, and tolerance to DNA damaging treatments. However, both wild-type ZMYM2 and the K529R mutant restored platinum-resistant phenotypes in ZMYM2-knockdown cells, indicating that K529 lactylation primarily maintains ZMYM2 stability rather than directly determining its downstream pro-resistance activity. Collectively, these findings identify a glycolysis–lactate–ZMYM2 lactylation axis that promotes platinum resistance in ovarian cancer and highlight lactylation-dependent ZMYM2 stabilization as a potential therapeutic vulnerability.

## 1. Introduction

Ovarian cancer remains one of the most lethal gynecologic malignancies and continues to impose a substantial global disease burden [1,2]. Due to its insidious onset and the lack of specific symptoms or effective early screening strategies, most patients are diagnosed at an advanced stage, resulting in poor long-term outcomes [3]. Among the histological subtypes, high-grade serous ovarian cancer (HGSOC) is the most common and aggressive form, accounting for the majority of advanced cases and ovarian cancer-related deaths [4,5,6]. Cytoreductive surgery combined with platinum-based chemotherapy remains the cornerstone of treatment for HGSOC [7]. Although many patients initially respond to platinum-based therapy, most eventually develop recurrent disease, and the emergence of platinum resistance remains a major cause of treatment failure and poor survival [8,9]. Therefore, elucidating the molecular mechanisms that drive and maintain platinum resistance is of substantial importance for improving therapeutic outcomes in ovarian cancer.

Platinum resistance in ovarian cancer is a multifactorial process involving diverse biological mechanisms, including enhanced DNA damage repair, epithelial–mesenchymal transition, altered cell survival signaling, and metabolic reprogramming [9,10]. Accumulating evidence indicates that metabolic reprogramming is closely involved in therapeutic resistance in cancer. In ovarian cancer, altered glucose metabolism has been increasingly linked to platinum resistance, and the glycolysis–lactate axis is now recognized as an important metabolic context for adaptive survival under treatment pressure [11,12]. Lactate is no longer viewed merely as a by-product of glycolysis, but rather as an active metabolite that can influence gene regulation, signaling pathways, and stress adaptation [13,14,15,16,17]. In 2019, Zhang et al. identified histone lysine lactylation as a lactate-derived post-translational modification that directly links glycolytic metabolism to chromatin regulation and gene expression [18]. Subsequent studies have further shown that lactylation extends beyond histones and may regulate protein function at the post-translational level [19,20,21,22,23]. Recent studies further suggest that lactylation can directly modulate DNA damage repair and treatment response. In particular, lactylation of DNA repair-associated proteins such as MRE11 and NBS1 has been shown to enhance homologous recombination (HR)-mediated repair [24,25]. In ovarian cancer, histone and RAD51 lactylation have been implicated in platinum resistance, and histone lactylation has also been linked to niraparib resistance [21,26]. These findings support the concept that protein lactylation may function as a molecular bridge through which aberrant glycolysis–lactate metabolism is translated into stable resistance-associated phenotypes. However, the key non-histone lactylation events that drive platinum resistance in ovarian cancer remain largely undefined.

Among the molecular processes that determine platinum responsiveness, DNA damage repair—particularly HR—is of central importance [27,28]. Platinum drugs exert cytotoxic effects primarily by inducing DNA lesions, and enhanced HR repair is a well-recognized mechanism by which tumor cells tolerate DNA damage and survive platinum treatment [29,30]. In this context, ZMYM2 is of particular interest. ZMYM2 is a nuclear zinc finger protein implicated in chromatin-associated regulatory processes, and previous work has shown that it restricts 53BP1 engagement at DNA double-strand breaks, facilitates BRCA1 loading, and promotes HR repair [31,32]. These findings suggest that ZMYM2 may act as a DNA repair-associated regulator with direct relevance to platinum responsiveness. However, whether ZMYM2 is subject to metabolically driven post-translational regulation, particularly lactylation, and whether such regulation contributes to ovarian cancer platinum resistance, has not been established. This knowledge gap provided the rationale for investigating ZMYM2 as a potential node linking lactate metabolism, protein lactylation, HR repair, and platinum resistance.

In this study, we sought to determine whether aberrant glycolysis–lactate metabolism promotes platinum resistance in ovarian cancer through protein lactylation and to identify the key effector mechanism underlying this process. By integrating clinical cohort analysis, proteomic and lactylomic profiling, and functional validation in ovarian cancer cell models, we identified ZMYM2 K529 lactylation as a platinum resistance-associated event. We further demonstrated that lactate-induced K529 lactylation suppresses ubiquitin–proteasome-mediated degradation of ZMYM2, thereby enhancing its protein stability and abundance. Functionally, elevated ZMYM2 promotes cisplatin tolerance, homologous recombination repair, and tolerance to DNA damage-inducing treatments. These findings uncover a glycolysis–lactate–ZMYM2 lactylation axis in ovarian cancer and provide mechanistic insight into how metabolic reprogramming contributes to platinum resistance.

## 2. Results

### 2.1. Platinum-Resistant Ovarian Cancer Cells Exhibit Enhanced Glycolysis, Lactate Accumulation, and Elevated Global Protein Lactylation

**Figure 1:**
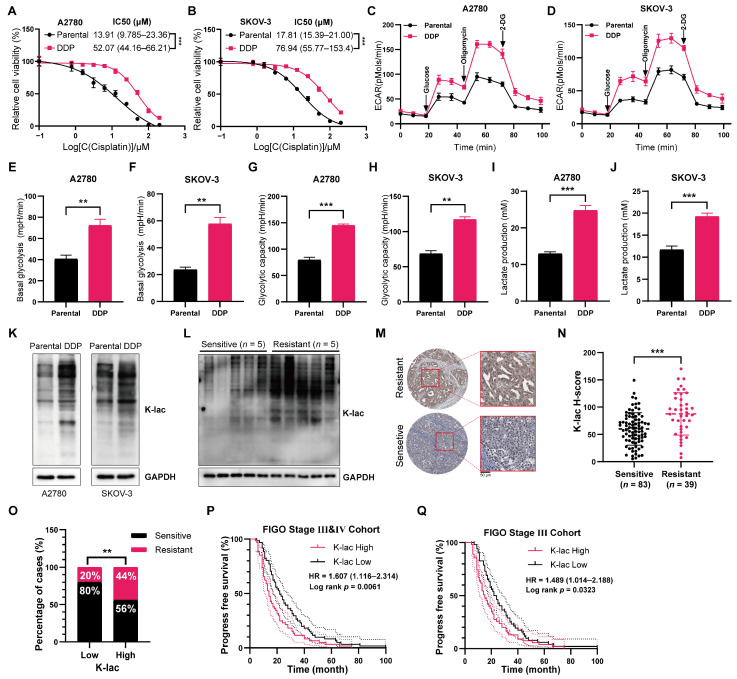
Platinum-resistant ovarian cancer cells exhibit enhanced glycolysis, lactate accumulation, and elevated global protein lactylation. (**A**,**B**) Cell viability and IC50 values of A2780/A2780-DDP (**A**) and SKOV-3/SKOV-3-DDP cells (**B**) following cisplatin treatment, as determined by the CCK-8 assays. (**C**–**H**) ECAR profiles of A2780/A2780-DDP (**C**) and SKOV-3/SKOV-3-DDP cells (**D**), with quantification of basal glycolysis and glycolytic capacity in A2780/A2780-DDP (**E**,**F**) and SKOV-3/SKOV-3-DDP cells (**G**,**H**). (**I**,**J**) Intracellular lactate levels in A2780/A2780-DDP (**I**) and SKOV-3/SKOV-3-DDP cells (**J**). (**K**,**L**) Global protein lactylation levels in ovarian cancer cell lines (**K**) and in platinum-sensitive (*n* = 5) and platinum-resistant (*n* = 5) ovarian cancer tissues (**L**), as determined by Western blotting. (**M**–**O**) Representative IHC images (**M**), quantitative K-lac H-scores (**N**), and association of K-lac levels with platinum sensitivity/resistance status (**O**) in ovarian cancer tissues. Scale bar: 50 μm. (**P**,**Q**) Kaplan–Meier analysis of progression-free survival (PFS) in the entire cohort (P) and the FIGO stage III subgroup (Q), stratified by K-lac H-scores. For all panels, *n* = 3 biological replicates per group unless otherwise indicated. Data are presented as mean ± SD. Statistical significance was determined by unpaired two-tailed Student’s *t*-test for comparisons between two groups, chi-square test for categorical association analysis, and log-rank test for Kaplan–Meier survival analysis. ** *p* < 0.01, *** *p* < 0.001.

To characterize the metabolic alterations associated with platinum resistance in ovarian cancer, we first assessed the cisplatin sensitivity of parental and resistant cell lines. CCK-8 assays showed that A2780-DDP and SKOV-3-DDP cells were less sensitive to cisplatin than their respective parental cells, as indicated by the rightward shift of the dose–response curves and increased half maximal inhibitory concentration (IC50) values (Figure 1A,B). These findings confirmed the platinum-resistant phenotype of the established cell models. We next examined whether platinum resistance was accompanied by glycolytic reprogramming. Extracellular acidification rate (ECAR) analysis revealed markedly increased glycolytic activity in A2780-DDP and SKOV-3-DDP cells relative to their parental counterparts (Figure 1C,D). Consistently, both basal glycolysis and glycolytic capacity were significantly elevated in resistant cells (Figure 1E–H). In line with these metabolic changes, intracellular lactate levels were also increased in A2780-DDP and SKOV-3-DDP cells (Figure 1I,J), indicating enhanced lactate accumulation under the platinum-resistant state.

Given that lactate serves as the metabolic substrate for protein lactylation, we further assessed the global protein lactylation levels in platinum-sensitive and platinum-resistant ovarian cancer models. Western blot analysis showed that global protein lactylation was increased in the A2780-DDP and SKOV-3-DDP cells compared with the corresponding parental cells (Figure 1K). A similar increase was observed in platinum-resistant ovarian cancer tissues relative to platinum-sensitive tissues (Figure 1L). To further assess the clinical relevance of global protein lactylation, we performed immunohistochemical analysis in a retrospective cohort of 122 patients with HGSOC, including 83 platinum-sensitive and 39 platinum-resistant cases. The clinicopathological characteristics of this cohort, including International Federation of Gynecology and Obstetrics (FIGO) stage distribution, are summarized in Table S1. Immunohistochemical staining (IHC) further confirmed stronger lactylation signals in platinum-resistant tumors (Figure 1M), and quantitative analysis demonstrated significantly higher protein lysine lactylation (K-lac) H-scores in the platinum-resistant group than in the platinum-sensitive group (Figure 1N). Moreover, elevated protein lactylation was significantly associated with platinum-resistant classification in ovarian cancer tissues (Figure 1O). Kaplan–Meier analysis of the 122-patient cohort showed that patients with high K-lac levels had shorter progression-free survival (PFS) than those with low K-lac levels (Figure 1P). This association was also observed in the FIGO stage III subgroup (Figure 1Q). Together, these findings indicate that platinum-resistant ovarian cancer is characterized by enhanced glycolysis, lactate accumulation, and elevated global protein lactylation, and that increased lactylation is associated with unfavorable clinical outcome.

### 2.2. Lactate Promotes, Whereas Glycolytic Inhibition Suppresses, Platinum Resistance in Ovarian Cancer Cells

**Figure 2:**
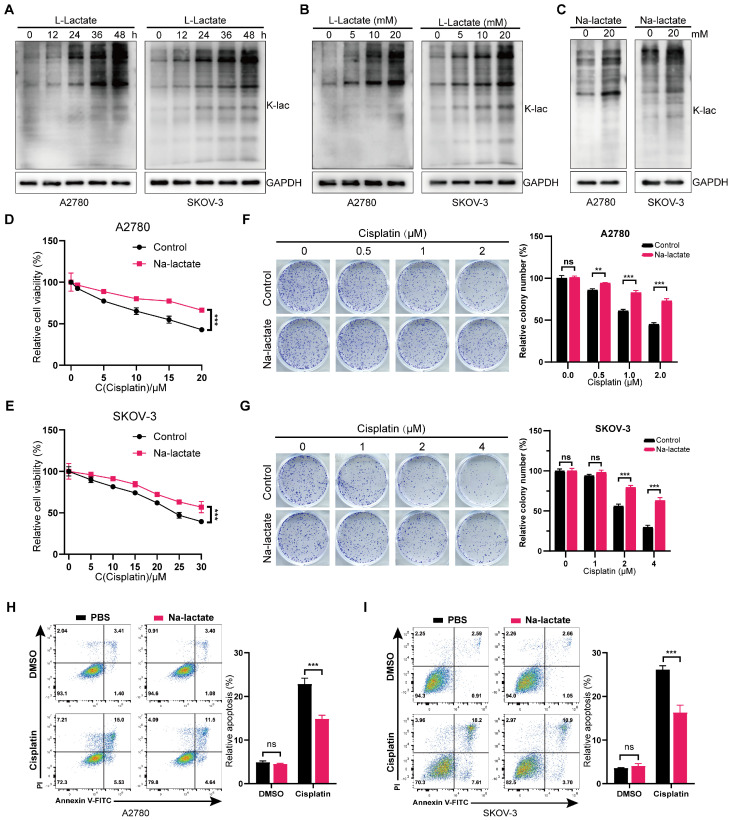
Exogenous lactate increases global protein lactylation and promotes platinum resistance in ovarian cancer cells. (**A**) Global protein lactylation levels in A2780 and SKOV-3 cells treated with 20 mM lactate for 0, 12, 24, 36, and 48 h, as detected by Western blotting. (**B**) Global protein lactylation levels in A2780 and SKOV-3 cells treated with increasing concentrations of lactate for 48 h, as detected by Western blotting. (**C**) Global protein lactylation levels in A2780 and SKOV-3 cells treated with sodium lactate for 48 h, as detected by Western blotting. (**D**,**E**) Cisplatin sensitivity of A2780 cells (**D**) and SKOV-3 cells (**E**) following treatment with 20 mM sodium lactate, as determined by CCK-8 assays. (**F**,**G**) Colony-forming ability of A2780 cells (**F**) and SKOV-3 cells (**G**) after cisplatin treatment in the presence of 20 mM sodium lactate, together with the quantification of colony numbers. (**H**,**I**) Apoptosis of A2780 cells (**H**) and SKOV-3 cells (**I**) following treatment with 10 μM cisplatin in the presence of 20 mM sodium lactate, as determined by Annexin V-FITC/PI staining and flow cytometry, together with the quantification of early and late apoptotic cells. For all panels, *n* = 3 biological replicates per group unless otherwise indicated. Data are presented as mean ± SD. Statistical significance was determined by two-way ANOVA for dose–response curves and by unpaired two-tailed Student’s *t*-test for comparisons between two groups. ns, no significance, ** *p* < 0.01, *** *p* < 0.001.

To determine whether lactate directly influences protein lactylation in ovarian cancer cells, we first examined the effects of exogenous lactate treatment on global protein lactylation levels. Western blot analysis showed that treatment with 20 mM lactate induced a time-dependent increase in global protein lactylation in both A2780 and SKOV-3 cells, with progressive elevation observed from 12 to 48 h (Figure 2A). Consistently, exposure to increasing concentrations of lactate for 48 h resulted in a dose-dependent increase in global protein lactylation in both cell lines (Figure 2B). Similar results were obtained following treatment with sodium lactate, which also markedly increased the global protein lactylation levels in A2780 and SKOV-3 cells (Figure 2C). These findings indicate that exogenous lactate is sufficient to enhance protein lactylation in ovarian cancer cells. We next investigated whether lactate-induced protein lactylation affects cisplatin responsiveness. CCK-8 assays showed that treatment with 20 mM sodium lactate decreased the sensitivity of both A2780 and SKOV-3 cells to cisplatin, as reflected by increased cell viability under cisplatin exposure (Figure 2D,E). In agreement with these results, colony formation assays demonstrated that sodium lactate significantly enhanced the clonogenic survival of both cell lines after cisplatin treatment (Figure 2F,G). Moreover, flow cytometric analysis revealed that sodium lactate reduced cisplatin-induced apoptosis in A2780 and SKOV-3 cells, as indicated by decreased proportions of early and late apoptotic cells (Figure 2H,I). Together, these results suggest that exogenous lactate promotes platinum resistance in ovarian cancer cells.

**Figure 3:**
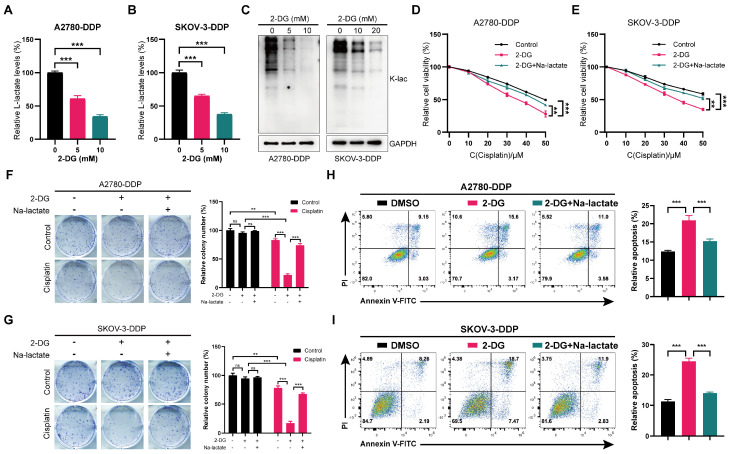
Glycolytic inhibition reduces lactate production and global protein lactylation and suppresses platinum resistance in ovarian cancer cells. (**A**,**B**) Intracellular lactate levels in A2780-DDP cells (**A**) and SKOV-3-DDP cells (**B**) treated with increasing concentrations of 2-DG. (**C**) Global protein lactylation levels in A2780-DDP and SKOV-3-DDP cells treated with increasing concentrations of 2-DG, as detected by Western blotting. (**D**,**E**) Cisplatin sensitivity of A2780-DDP cells (**D**) and SKOV-3-DDP cells (**E**) treated with 10 mM 2-DG with or without supplementation of 20 mM sodium lactate, as determined by CCK-8 assays. (**F**,**G**) Colony-forming ability of A2780-DDP cells (**F**) and SKOV-3-DDP cells (**G**) following treatment with 5 μM cisplatin in the presence of 10 mM 2-DG with or without supplementation of 20 mM sodium lactate, together with the quantification of colony numbers. (**H**,**I**) Apoptosis of A2780-DDP cells (**H**) and SKOV-3-DDP cells (**I**) following treatment with 20 μM cisplatin in the presence of 10 mM 2-DG with or without supplementation of 20 mM sodium lactate, as determined by Annexin V-FITC/PI staining and flow cytometry, together with the quantification of apoptotic cells. For all panels, *n* = 3 biological replicates per group unless otherwise indicated. Data are presented as mean ± SD. Statistical significance was determined by one-way ANOVA for comparisons among multiple groups and by two-way ANOVA for dose–response curves, as appropriate. ns, no significance, ** *p* < 0.01, *** *p* < 0.001.

To further determine whether glycolytic activity contributes to lactate accumulation and platinum resistance, we inhibited glycolysis in platinum-resistant cells using 2-deoxy-D-glucose (2-DG). Treatment with increasing concentrations of 2-DG reduced the intracellular lactate levels in both A2780-DDP and SKOV-3-DDP cells in a dose-dependent manner (Figure 3A,B). Consistently, the global protein lactylation levels were also decreased following 2-DG treatment in both resistant cell lines (Figure 3C), indicating that the inhibition of glycolysis suppresses lactate production and downstream protein lactylation. We then assessed the functional consequences of glycolytic inhibition on cisplatin sensitivity. CCK-8 assays showed that 2-DG treatment significantly increased the sensitivity of A2780-DDP and SKOV-3-DDP cells to cisplatin, whereas supplementation with 20 mM sodium lactate partially reversed this effect (Figure 3D,E). Similar findings were obtained in the colony formation assays, in which 2-DG markedly reduced clonogenic survival after cisplatin treatment, while sodium lactate supplementation partially restored the colony-forming ability (Figure 3F,G). In addition, flow cytometric analysis demonstrated that 2-DG enhanced cisplatin-induced apoptosis in both resistant cell lines, and this pro-apoptotic effect was partially attenuated by sodium lactate supplementation (Figure 3H,I). Collectively, these findings indicate that lactate promotes, whereas glycolytic inhibition suppresses, platinum resistance in ovarian cancer cells, supporting a functional role for the glycolysis–lactate axis in modulating cisplatin responsiveness.

### 2.3. Lactylome Profiling Identifies ZMYM2 K529 Lactylation as a Candidate Event Associated with Platinum Resistance

**Figure 4:**
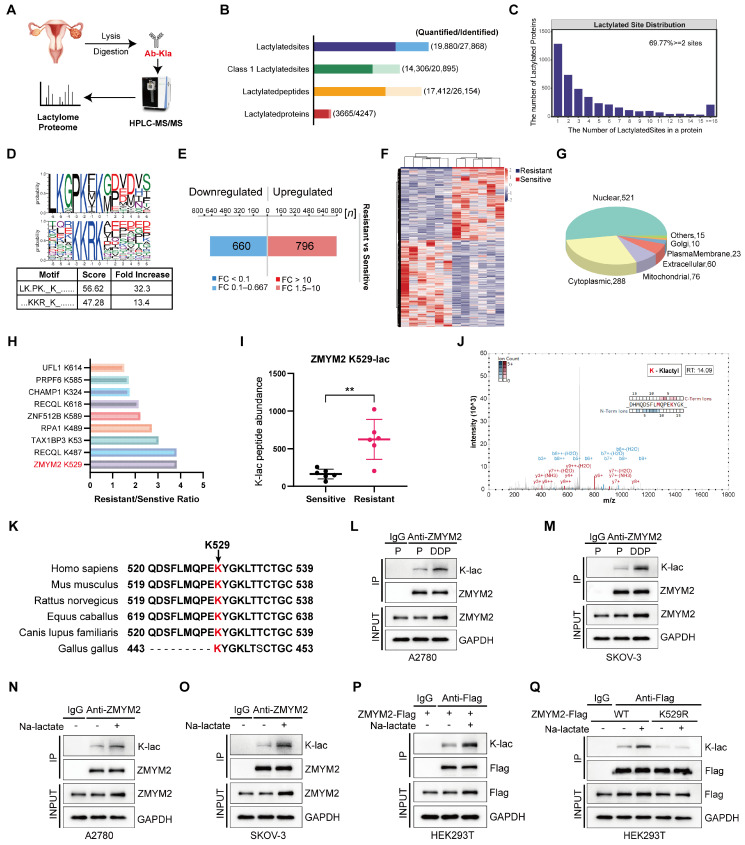
Lactylome profiling identified ZMYM2 K529 lactylation as a candidate event associated with platinum resistance. (**A**) Schematic workflow of the proteomic and lactylomic analyses. (**B**,**C**) Summary of identified lactylation sites, Class I sites, lactylated peptides, and lactylated proteins (**B**), and distribution of lactylation sites per protein (**C**). (**D**) Motif enrichment analysis of all identified lactylated peptides showing the top two enriched conserved motifs. (**E**,**F**) Distribution of upregulated and downregulated lactylated peptides (**E**) and hierarchical clustering of differentially lactylated peptides (**F**) in platinum-resistant versus platinum-sensitive ovarian cancer tissues. (**G**) Subcellular localization analysis of differentially lactylated proteins. (**H**) Nuclear-localized candidate proteins showing significant differential lactylation in the lactylome analysis. (**I**) Quantification of ZMYM2 K529 lactylation levels in platinum-resistant (*n* = 6) and platinum-sensitive (*n* = 6) ovarian cancer tissues. (**J**) Representative LC–MS/MS spectrum identifying lactylation at the ZMYM2 K529 site. (**K**) Conservation analysis of the ZMYM2 amino acid sequence across six species. (**L**,**M**) ZMYM2 lactylation levels in parental and platinum-resistant A2780 cells (**L**) and SKOV-3 cells (**M**), as determined by immunoprecipitation followed by Western blotting. (**N**,**O**) ZMYM2 lactylation levels in A2780 cells (**N**) and SKOV-3 cells (**O**) following treatment with 20 mM sodium lactate, as determined by immunoprecipitation followed by Western blotting. (**P**) Lactylation of ectopically expressed ZMYM2-Flag in HEK293T cells following treatment with 20 mM sodium lactate, as determined by immunoprecipitation followed by Western blotting. (**Q**) Comparison of lactylation levels between ZMYM2-WT-Flag and ZMYM2-K529R-Flag in HEK293T cells following treatment with 20 mM sodium lactate, as determined by immunoprecipitation followed by Western blotting. For all panels, *n* = 3 biological replicates per group unless otherwise indicated. Data are presented as mean ± SD. Statistical significance was determined by unpaired two-tailed Student’s *t*-test for comparisons between two groups. ** *p* < 0.01.

Given that platinum-resistant ovarian cancer exhibited elevated global protein lactylation, we next sought to identify specific lactylated proteins that might contribute to the platinum-resistant phenotype. To this end, we performed integrated proteomic and lactylomic analyses using fresh-frozen tumor tissues from 12 patients with high-grade serous ovarian cancer, including 6 platinum-resistant and 6 platinum-sensitive cases (Figure 4A). Global lactylome profiling revealed a broad lactylation landscape in ovarian cancer tissues, including numerous lactylation sites, Class I sites, lactylated peptides, and modified proteins (Figure 4B). Here, Class I sites refer to lactylation sites with high-confidence localization. Site distribution analysis showed that most proteins contained one or a limited number of lactylation sites (Figure 4C), whereas motif enrichment analysis identified the top two significantly enriched conserved motifs surrounding lactylated lysine residues (Figure 4D). Quantitative lactylome analysis further identified 1456 differentially lactylated peptides between platinum-resistant and platinum-sensitive ovarian cancer tissues, including 796 upregulated and 660 downregulated peptides (Figure 4E,F). Subcellular localization analysis showed that a substantial proportion of differentially lactylated proteins were localized to the nucleus (Figure 4G), supporting the possibility that nuclear lactylated proteins may contribute to platinum resistance.

Together, these findings suggest broad remodeling of the lactylation landscape in platinum-resistant ovarian cancer tissues, particularly involving nuclear-localized proteins. We therefore focused on nuclear-localized proteins with significant differential lactylation to identify candidate events with potential mechanistic relevance to platinum resistance (Figure 4H). Among these candidates, ZMYM2 was selected for further investigation based on fold change, statistical significance, and subcellular localization. Quantitative lactylome analysis showed that lactylation at the ZMYM2 K529 site was significantly increased in platinum-resistant ovarian cancer tissues compared with platinum-sensitive tissues, with a fold change of 3.828 and a *p* value of 0.0071 (Figure 4I). Liquid chromatography-tandem mass spectrometry (LC-MS/MS) analysis further confirmed lactylation at lysine 529 of ZMYM2 (Figure 4J). Sequence alignment additionally showed that the K529-containing region of ZMYM2 was highly conserved across multiple species (Figure 4K), suggesting potential functional relevance of this site. We then validated ZMYM2 lactylation at the cellular level. Immunoprecipitation followed by Western blotting showed that ZMYM2 lactylation was increased in platinum-resistant A2780 and SKOV-3 cells compared with their corresponding parental cells (Figure 4L,M). Consistently, treatment with 20 mM sodium lactate further enhanced ZMYM2 lactylation in both A2780 and SKOV-3 cells (Figure 4N,O). In HEK293T cells transiently expressing ZMYM2-Flag, sodium lactate treatment similarly increased the lactylation signal of ZMYM2 (Figure 4P). Notably, mutation of lysine 529 to arginine (K529R) markedly attenuated the lactylation level of ZMYM2 in the presence of sodium lactate (Figure 4Q), supporting the notion that K529 is a major lactylation site of ZMYM2. Collectively, these findings identify ZMYM2 K529 lactylation as a candidate molecular event associated with platinum resistance in ovarian cancer.

### 2.4. ZMYM2 Is Upregulated in Platinum-Resistant Ovarian Cancer and Associated with Adverse Clinical Features

**Figure 5:**
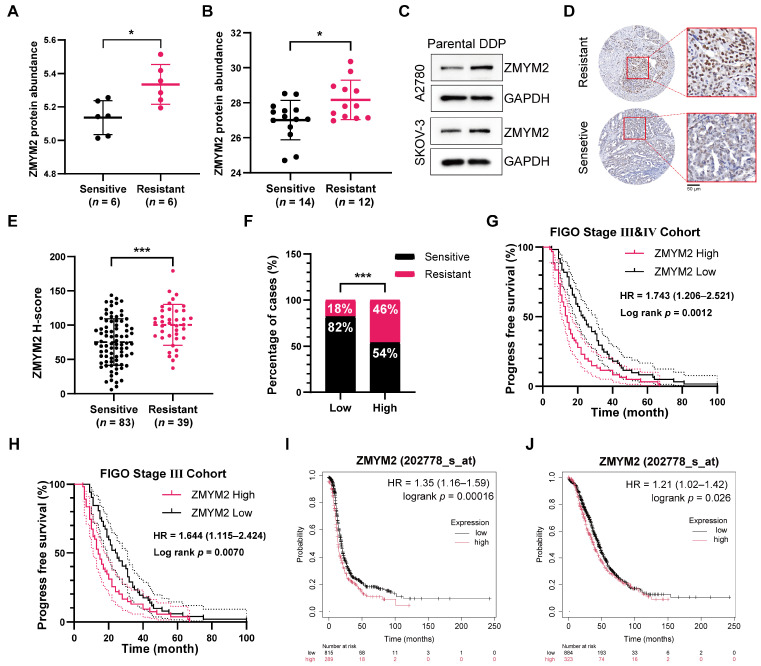
ZMYM2 is upregulated in platinum-resistant ovarian cancer and is associated with adverse clinical features. (**A**) Differential ZMYM2 protein expression between platinum-sensitive (*n* = 6) and platinum-resistant (*n* = 6) ovarian cancer tissues based on the proteomic data generated in this study. (**B**) Differential ZMYM2 protein expression between platinum-sensitive (*n* = 14) and platinum-resistant (*n* = 12) ovarian cancer tissues based on a public proteomic dataset. (**C**) ZMYM2 protein expression in parental ovarian cancer cells and their corresponding platinum-resistant cells, as determined by Western blotting. (**D**–**F**) Representative IHC images of ZMYM2 expression (**D**), quantitative ZMYM2 H-scores (**E**), and association of ZMYM2 expression with platinum sensitivity/resistance status (**F**) in ovarian cancer tissues. (**G**,**H**) Kaplan–Meier analysis of progression-free survival (PFS) in the entire cohort (**G**) and the FIGO stage III subgroup (**H**), stratified by ZMYM2 H-scores. (**I**,**J**) Kaplan–Meier Plotter analysis of the association between ZMYM2 mRNA expression and PFS (**I**) or OS (**J**) in ovarian cancer patients. For all panels, *n* = 3 biological replicates per group unless otherwise indicated. Data are presented as mean ± SD. Statistical significance was determined by unpaired two-tailed Student’s *t*-test for comparisons between two groups, chi-square test for categorical association analysis, and log-rank test for Kaplan–Meier survival analysis. * *p* < 0.05, *** *p* < 0.001.

To further determine the clinical relevance of ZMYM2 in platinum-resistant ovarian cancer, we first examined its expression in ovarian cancer tissues. Analysis of the proteomic data generated in this study showed that ZMYM2 protein levels were increased in platinum-resistant ovarian cancer tissues compared with platinum-sensitive tissues (Figure 5A). This finding was further supported by the analysis of a public proteomic dataset [33] that likewise showed higher ZMYM2 protein expression in platinum-resistant ovarian cancer tissues than in platinum-sensitive tissues (Figure 5B). Consistently, Western blot analysis demonstrated that ZMYM2 protein expression was elevated in platinum-resistant A2780-DDP and SKOV-3-DDP cells relative to their respective parental cells (Figure 5C). These results indicate that ZMYM2 is upregulated in platinum-resistant ovarian cancer at both the tissue and cellular levels. We next assessed ZMYM2 expression in the retrospective clinical cohort by immunohistochemistry. Representative staining images showed stronger ZMYM2 signals in platinum-resistant ovarian cancer tissues than in platinum-sensitive tissues (Figure 5D). Quantitative analysis further demonstrated that ZMYM2 H-scores were significantly higher in the platinum-resistant group than in the platinum-sensitive group (Figure 5E). In addition, elevated ZMYM2 expression was significantly associated with platinum-resistant classification in ovarian cancer tissues (Figure 5F). To evaluate the prognostic significance of ZMYM2, Kaplan–Meier analysis was performed in the 122-patient cohort. Patients with high ZMYM2 expression exhibited shorter progression-free survival (PFS) than those with low ZMYM2 expression (Figure 5G). A similar association was observed in the FIGO stage III subgroup (Figure 5H). Moreover, analysis using the Kaplan–Meier Plotter database showed that higher ZMYM2 mRNA expression was associated with worse PFS and overall survival (OS) in ovarian cancer patients (Figure 5I,J). Collectively, these findings indicate that ZMYM2 is upregulated in platinum-resistant ovarian cancer and is associated with platinum-resistant phenotype and unfavorable prognosis.

### 2.5. Lactate Stabilizes ZMYM2 by Promoting K529 Lactylation and Suppressing Ubiquitin–Proteasome-Mediated Degradation

**Figure 6:**
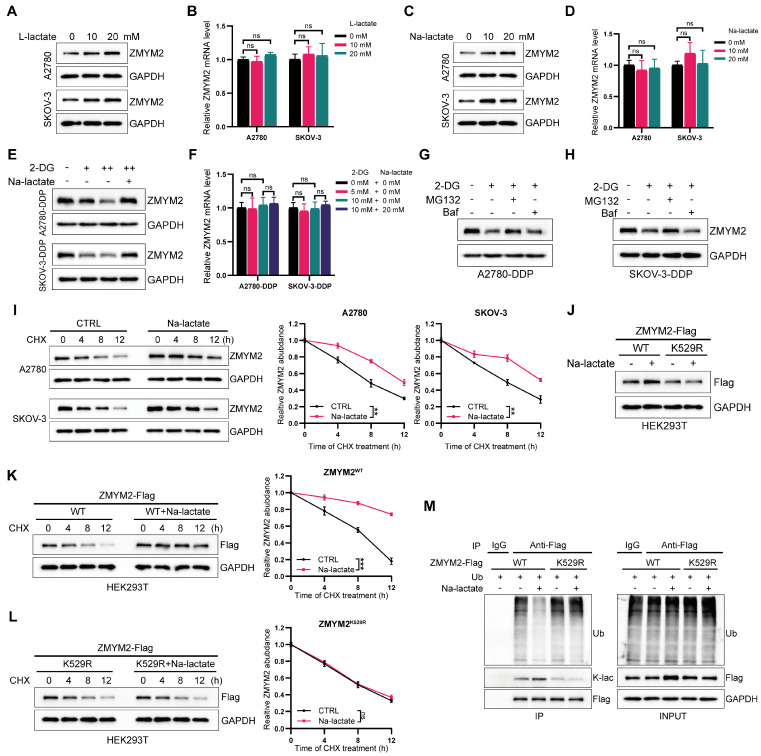
Lactate stabilizes ZMYM2 by promoting K529 lactylation and suppressing ubiquitin–proteasome-mediated degradation. (**A**–**D**) ZMYM2 protein and mRNA levels in A2780 and SKOV-3 cells treated with increasing concentrations of lactate (**A**,**B**) or sodium lactate (**C**,**D**), as determined by Western blotting and qRT-PCR. (**E**,**F**) ZMYM2 protein and mRNA levels in A2780-DDP and SKOV-3-DDP cells treated with 2-DG with or without sodium lactate. (**G**,**H**) ZMYM2 protein levels in A2780-DDP (**G**) and SKOV-3-DDP cells (**H**) treated with 2-DG alone or in combination with MG132 or Bafilomycin A1, as determined by Western blotting. (**I**) Effects of sodium lactate on endogenous ZMYM2 protein stability in ovarian cancer cells treated with CHX, together with densitometric quantification. (**J**) ZMYM2 protein levels in HEK293T cells transiently expressing ZMYM2-WT-Flag or ZMYM2-K529R-Flag following sodium lactate treatment. (**K**,**L**) Effects of sodium lactate on the stability of ectopically expressed ZMYM2-WT-Flag (**K**) and ZMYM2-K529R-Flag (**L**) in HEK293T cells treated with CHX, together with densitometric quantification. (**M**) Lactylation and ubiquitination levels of wild-type ZMYM2 and the ZMYM2-K529R mutant in HEK293T cells treated with sodium lactate, as determined by immunoprecipitation followed by Western blotting. For all panels, *n* = 3 biological replicates per group unless otherwise indicated. Data are presented as mean ± SD. Statistical significance was determined by unpaired two-tailed Student’s *t*-test for comparisons between two groups, one-way ANOVA for comparisons among multiple groups, and two-way ANOVA for CHX chase/time-course analyses, as appropriate. ns, no significance, ** *p* < 0.01, *** *p* < 0.001.

Given that ZMYM2 K529 lactylation and ZMYM2 protein expression were both increased in platinum-resistant ovarian cancer, we next investigated whether lactate-induced K529 lactylation might regulate ZMYM2 protein abundance. To address this question, we first examined the effects of exogenous lactate on the ZMYM2 protein and mRNA levels in ovarian cancer cells. Western blot analysis showed that lactate treatment increased ZMYM2 protein expression in A2780 and SKOV-3 cells in a dose-dependent manner (Figure 6A). Similar results were obtained following sodium lactate treatment (Figure 6C). In contrast, quantitative reverse transcription-polymerase chain reaction (qRT-PCR) analysis showed no obvious change in ZMYM2 mRNA levels under either lactate or sodium lactate treatment (Figure 6B,D), indicating that lactate regulates ZMYM2 primarily at the protein level rather than at the transcriptional level. Consistently, the inhibition of glycolysis with 2-DG reduced the ZMYM2 protein expression in A2780-DDP and SKOV-3-DDP cells, whereas sodium lactate supplementation partially restored the ZMYM2 protein levels (Figure 6E). However, these treatments had little effect on ZMYM2 mRNA expression (Figure 6F). Together, these findings suggest that the glycolysis–lactate axis regulates ZMYM2 mainly through post-transcriptional mechanisms. We next investigated whether lactate affects ZMYM2 protein stability. In platinum-resistant ovarian cancer cells, the decrease in ZMYM2 induced by 2-DG was largely reversed by the proteasome inhibitor MG132, but not by the lysosomal inhibitor Bafilomycin A1 (Figure 6G,H), suggesting that glycolysis–lactate metabolism regulates ZMYM2 predominantly through the ubiquitin–proteasome pathway. Consistent with this, cycloheximide (CHX) chase assays showed that sodium lactate treatment prolonged the half-life of endogenous ZMYM2 in ovarian cancer cells (Figure 6I), indicating that lactate enhances ZMYM2 protein stability.

To further determine whether K529 lactylation contributes to ZMYM2 stabilization, we compared the effects of sodium lactate on wild-type ZMYM2 and the K529R mutant. In HEK293T cells, sodium lactate increased the protein level of ectopically expressed ZMYM2-WT-Flag, whereas this effect was attenuated in cells expressing ZMYM2-K529R-Flag (Figure 6J). CHX chase assays further showed that sodium lactate markedly prolonged the half-life of ZMYM2-WT-Flag, whereas the stabilizing effect was substantially weakened in the K529R mutant (Figure 6K,L). Immunoprecipitation followed by Western blotting demonstrated that sodium lactate increased lactylation and reduced the ubiquitination of wild-type ZMYM2, whereas the K529R mutation markedly impaired lactylation and attenuated the inhibitory effect of sodium lactate on ZMYM2 ubiquitination (Figure 6M). Collectively, these findings, together with the preceding results showing enhanced glycolysis and lactate accumulation in platinum-resistant ovarian cancer, indicate that lactate promotes ZMYM2 K529 lactylation, suppresses ubiquitin–proteasome-mediated degradation, and thereby increases ZMYM2 protein stability, contributing to the maintenance of elevated ZMYM2 expression.

### 2.6. ZMYM2 Promotes Platinum-Resistant Phenotypes in Ovarian Cancer

**Figure 7:**
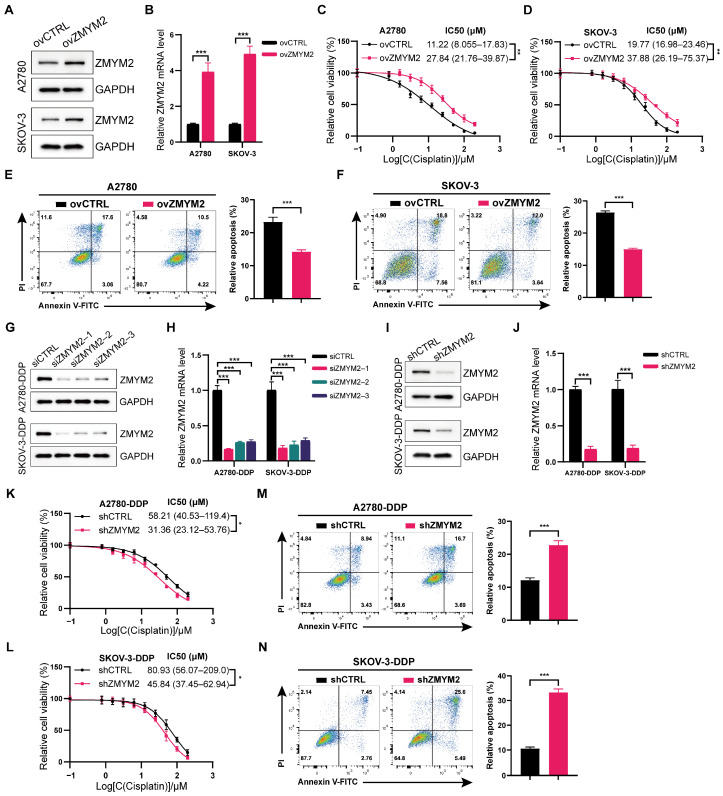
ZMYM2 promotes platinum-resistant phenotypes in ovarian cancer cells. (**A**,**B**) Validation of stable ZMYM2 overexpression in ovarian cancer cells by Western blotting (**A**) and qRT-PCR (**B**). (**C**,**D**) Cell viability and IC50 values of A2780 ovCTRL/A2780 ovZMYM2 (**C**) and SKOV-3 ovCTRL/SKOV-3 ovZMYM2 cells (**D**) following cisplatin treatment, as determined by CCK-8 assays. (**E**,**F**) Apoptosis of A2780 ovCTRL/A2780 ovZMYM2 (**E**) and SKOV-3 ovCTRL/SKOV-3 ovZMYM2 cells (**F**) after cisplatin treatment, as determined by Annexin V-FITC/PI staining and flow cytometry, together with quantitative analysis. (**G**,**H**) Validation of siRNA-mediated ZMYM2 knockdown in platinum-resistant ovarian cancer cells by Western blotting (**G**) and qRT-PCR (**H**). (**I**,**J**) Validation of stable ZMYM2 knockdown in platinum-resistant ovarian cancer cells by Western blotting (**I**) and qRT-PCR (**J**). (**K**,**L**) Cell viability and IC50 values of A2780-DDP shCTRL/A2780-DDP shZMYM2 (**K**) and SKOV-3-DDP shCTRL/SKOV-3-DDP shZMYM2 cells (**L**) following cisplatin treatment, as determined by CCK-8 assays. (**M**,**N**) Apoptosis of A2780-DDP shCTRL/A2780-DDP shZMYM2 (**M**) and SKOV-3-DDP shCTRL/SKOV-3-DDP shZMYM2 cells (**N**) after cisplatin treatment, as determined by Annexin V-FITC/PI staining and flow cytometry, together with quantitative analysis. For all panels, *n* = 3 biological replicates per group unless otherwise indicated. Data are presented as mean ± SD. Statistical significance was determined by unpaired two-tailed Student’s *t*-test for comparisons between two groups and by two-way ANOVA for dose–response curves, as appropriate. * *p* < 0.05, ** *p* < 0.01, *** *p* < 0.001.

Given that ZMYM2 was upregulated in platinum-resistant ovarian cancer and associated with adverse clinical features, and that lactate-induced K529 lactylation increased ZMYM2 stability and expression, we next investigated whether elevated ZMYM2 functionally promotes platinum-resistant phenotypes in ovarian cancer cells. Stable overexpression of ZMYM2 in A2780 and SKOV-3 cells was confirmed by Western blotting and qRT-PCR (Figure 7A,B). CCK-8 assays showed that ZMYM2 overexpression reduced the sensitivity of both cell lines to cisplatin, as reflected by rightward shifts in the dose–response curves and increased IC50 values (Figure 7C,D). Consistently, flow cytometric analysis demonstrated that ZMYM2 overexpression decreased cisplatin-induced apoptosis in both A2780 and SKOV-3 cells (Figure 7E,F). These findings indicate that ZMYM2 overexpression is sufficient to enhance platinum tolerance in ovarian cancer cells. To further determine whether endogenous ZMYM2 is required for maintenance of the resistant phenotype, we first validated siRNA-mediated ZMYM2 knockdown in platinum-resistant ovarian cancer cells (Figure 7G,H), and then established stable ZMYM2-depleted A2780-DDP and SKOV-3-DDP cell lines (Figure 7I,J). CCK-8 assays showed that stable knockdown of ZMYM2 significantly increased the sensitivity of both resistant cell lines to cisplatin, as indicated by decreased cell viability and reduced IC50 values (Figure 7K,L). In parallel, Annexin V-FITC/PI staining showed that ZMYM2 depletion markedly enhanced cisplatin-induced apoptosis in A2780-DDP and SKOV-3-DDP cells (Figure 7M,N). Together, these results indicate that ZMYM2 is required for the maintenance of platinum-resistant phenotypes in ovarian cancer cells.

**Figure 8:**
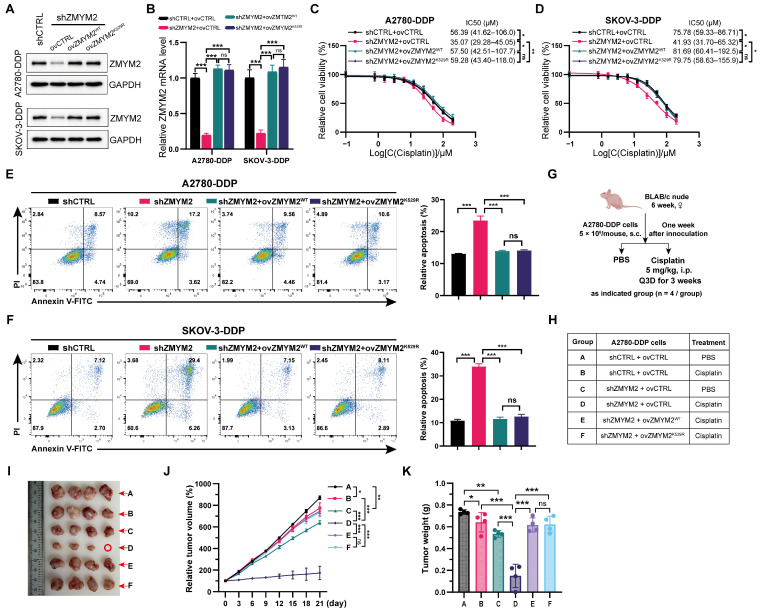
Rescue expression of either wild-type ZMYM2 or the K529R mutant restores platinum-resistant phenotypes in ZMYM2-knockdown cells. (**A**,**B**) Validation of rescue expression in platinum-resistant ovarian cancer cells with stable ZMYM2 knockdown followed by re-expression of the empty vector, ZMYM2-WT, or ZMYM2-K529R, as determined by Western blotting (**A**) and qRT-PCR (**B**). (**C**,**D**) Cell viability and IC50 values of A2780-DDP shZMYM2 (**C**) and SKOV-3-DDP shZMYM2 cells (**D**) after rescue expression of the empty vector, ZMYM2-WT, or ZMYM2-K529R and cisplatin treatment, as determined by CCK-8 assays. (**E**,**F**) Apoptosis of A2780-DDP shZMYM2 (**E**) and SKOV-3-DDP shZMYM2 cells (**F**) after rescue expression of the empty vector, ZMYM2-WT, or ZMYM2-K529R and cisplatin treatment, as determined by Annexin V-FITC/PI staining and flow cytometry, together with quantitative analysis. (**G**) Schematic diagram of the subcutaneous xenograft experiment using A2780-DDP cells. (**H**) Experimental grouping and treatment schedule for the xenograft study. (**I**) Representative images of xenograft tumors from each experimental group. (**J**) Tumor growth curves of xenografts in each group. (**K**) Final tumor weights in each group at the experimental endpoint. For all panels, *n* = 3 biological replicates per group unless otherwise indicated. Data are presented as mean ± SD. Statistical significance was determined by one-way ANOVA for comparisons among multiple groups, two-way ANOVA for dose–response curves and tumor growth curves, as appropriate. ns, no significance, * *p* < 0.05, ** *p* < 0.01, *** *p* < 0.001.

We next asked whether K529 lactylation directly determines the downstream pro-resistance activity of ZMYM2. To address this, rescue cell models were generated in the ZMYM2 knockdown background by re-expressing empty vector, ZMYM2-WT, or the ZMYM2-K529R mutant (Figure 8A,B). CCK-8 assays showed that re-expression of either ZMYM2-WT or ZMYM2-K529R restored cisplatin tolerance in both A2780-DDP shZMYM2 and SKOV-3-DDP shZMYM2 cells, whereas the empty vector did not (Figure 8C,D). Similarly, both ZMYM2-WT and ZMYM2-K529R reduced cisplatin-induced apoptosis in ZMYM2-depleted cells (Figure 8E,F). These findings suggest that once ZMYM2 expression is restored, K529 lactylation is not required for the downstream pro-resistance activity of ZMYM2. Finally, the role of ZMYM2 in platinum resistance was further examined in vivo using a subcutaneous xenograft model established with A2780-DDP cells (Figure 8G,H). Representative tumor images, tumor growth curves, and endpoint tumor weights consistently showed that ZMYM2 promoted tumor resistance to cisplatin treatment in vivo (Figure 8I–K). Collectively, these findings demonstrate that ZMYM2 promotes platinum-resistant phenotypes in ovarian cancer both in vitro and in vivo.

### 2.7. ZMYM2 Enhances Homologous Recombination Repair and Tolerance to DNA Damage in Ovarian Cancer Cells

Previous studies have suggested that ZMYM2 is involved in the regulation of DNA double-strand break repair and HR [32]. Because platinum drugs primarily exert their antitumor effects by inducing DNA damage, and HR is a major pathway for repairing DNA double-strand breaks, dysregulation of HR is closely linked to platinum responsiveness. In the present study, we found that lactate promoted ZMYM2 K529 lactylation and increased ZMYM2 protein stability, while elevated ZMYM2 enhanced platinum-resistant phenotypes in ovarian cancer cells. We therefore next asked whether lactylation-mediated stabilization of ZMYM2 could be translated into enhanced HR repair capacity and increased tolerance to DNA damage, and whether K529 lactylation directly contributes to these downstream functions.

**Figure 9:**
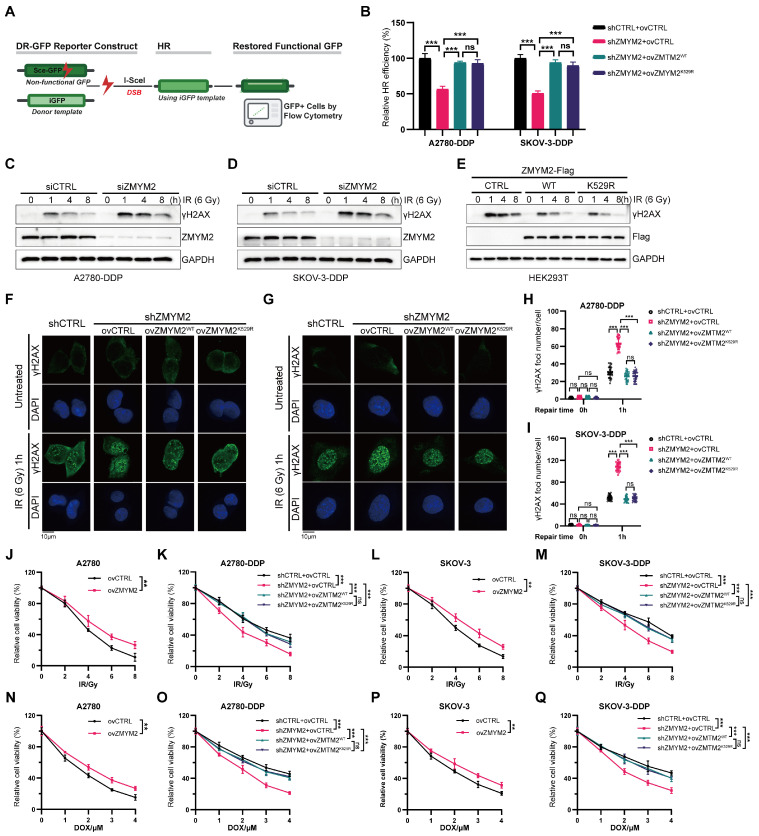
ZMYM2 enhances homologous recombination repair and tolerance to DNA damage in ovarian cancer cells. (**A**) Schematic illustration of the DR-GFP reporter system used to assess HR repair efficiency. (**B**) HR repair efficiency in the indicated platinum-resistant ovarian cancer cells, determined by flow cytometric quantification of GFP-positive cells after co-transfection of the I-SceI expression plasmid and the DR-GFP reporter construct. (**C**–**E**) γH2AX protein levels after IR, as determined by Western blotting, in control and transiently ZMYM2-depleted A2780-DDP (**C**) and SKOV-3-DDP cells (**D**), and in HEK293T cells overexpressing the empty vector, ZMYM2-WT, or ZMYM2-K529R (**E**). (**F**–**I**) Immunofluorescence analysis of γH2AX foci in the indicated stable A2780-DDP (**F**,**H**) and SKOV-3-DDP (**G**,**I**) cells at 0 h and 1 h after 6 Gy IR, together with the quantification of γH2AX foci numbers. Thirty cells were analyzed for each group. (**J**–**M**) Cell viability of the indicated stable ovarian cancer cell lines after exposure to the indicated doses of IR for 24 h, as determined by CCK-8 assays. (**N**–**Q**) Cell viability of the indicated stable ovarian cancer cell lines after treatment with the indicated concentrations of DOX for 24 h, as determined by CCK-8 assays. For all panels, *n* = 3 biological replicates per group unless otherwise indicated. Data are presented as mean ± SD. Statistical significance was determined by unpaired two-tailed Student’s *t*-test for comparisons between two groups, one-way ANOVA for comparisons among multiple groups, and two-way ANOVA for dose–response or time-course analyses, as appropriate. ns, no significance, ** *p* < 0.01, *** *p* < 0.001.

To address this question, we first assessed HR repair efficiency using the DR-GFP reporter system (Figure 9A). Flow cytometric analysis of GFP-positive cells showed that ZMYM2 depletion reduced HR repair efficiency in platinum-resistant ovarian cancer cells (Figure 9B), indicating that ZMYM2 contributes to HR-mediated DNA repair. We next examined the dynamic changes in γH2AX after ionizing radiation (IR). In both A2780-DDP and SKOV-3-DDP cells, transient knockdown of ZMYM2 altered the post-irradiation γH2AX profile, consistent with impaired DNA damage repair (Figure 9C,D). Conversely, overexpression of ZMYM2-WT or ZMYM2-K529R in HEK293T cells reduced the γH2AX levels after IR treatment compared with the empty-vector control (Figure 9E), suggesting that elevated ZMYM2 facilitates the resolution of DNA damage signaling. To further evaluate DNA repair capacity, we examined γH2AX foci formation in stable rescue models. In both the A2780-DDP and SKOV-3-DDP cells, ZMYM2 knockdown increased the number of residual γH2AX foci after IR, whereas re-expression of either ZMYM2-WT or ZMYM2-K529R reduced γH2AX foci accumulation (Figure 9F–I). These findings indicate that ZMYM2 enhances DNA damage repair capacity, and that restoration of ZMYM2 expression is sufficient to rescue this defect even when the K529 lactylation site is mutated.

We then assessed whether ZMYM2 affects cellular tolerance to DNA damage-inducing treatments. In parental A2780 and SKOV-3 cells, stable overexpression of ZMYM2 increased cell viability after IR exposure (Figure 9J,L) and after doxorubicin (DOX) treatment (Figure 9N,P), indicating enhanced tolerance to DNA damage. In platinum-resistant A2780-DDP and SKOV-3-DDP cells, ZMYM2 depletion reduced viability after IR and DOX treatment, whereas re-expression of either ZMYM2-WT or ZMYM2-K529R restored treatment tolerance (Figure 9K,M,O,Q). Collectively, these findings demonstrate that ZMYM2 enhances HR repair and promotes tolerance to DNA damage in ovarian cancer cells. Together with the results of the previous sections, these data further support the notion that K529 lactylation primarily contributes to platinum resistance by increasing ZMYM2 protein stability and abundance, rather than by directly determining its downstream DNA repair-promoting activity.

## 3. Discussion

In the present study, we identified a previously unrecognized link between aberrant glycolysis–lactate metabolism and platinum resistance in ovarian cancer. We show that platinum-resistant ovarian cancer is characterized by enhanced glycolysis, increased lactate accumulation, and elevated global protein lactylation, and further demonstrate that ZMYM2 K529 lactylation represents a functionally relevant event within this metabolic context. Mechanistically, lactate promoted ZMYM2 K529 lactylation and stabilized ZMYM2 by suppressing ubiquitin–proteasome-mediated degradation, thereby maintaining its elevated protein abundance. Functionally, increased ZMYM2 expression enhanced cisplatin tolerance, homologous recombination repair, and tolerance to DNA damage-inducing treatments. Together, these findings support a model in which lactate-driven ZMYM2 lactylation links metabolic reprogramming to platinum resistance and identify lactylation-dependent ZMYM2 stabilization as a potential therapeutic vulnerability in ovarian cancer.

Our findings should be interpreted in the context of growing evidence that metabolic reprogramming is closely associated with therapeutic resistance in ovarian cancer. Enhanced glycolysis and lactate accumulation have been increasingly linked to platinum resistance, supporting the idea that the glycolysis–lactate axis provides a metabolic basis for tumor cell survival under treatment pressure [14,34,35]. In parallel, lysine lactylation has emerged as a metabolite-derived post-translational modification that offers a direct mechanistic link between lactate metabolism and functional protein regulation [23,36,37]. Recent studies have further shown that lactylation can influence DNA damage repair and treatment response, including lactylation-dependent regulation of DNA repair-associated proteins and resistance-related pathways in cancer [24,25,38,39,40]. Consistent with these observations, our data showed that platinum-resistant ovarian cancer exhibited enhanced glycolysis, increased lactate accumulation, and elevated global protein lactylation, and that metabolic intervention by exogenous lactate supplementation or glycolysis inhibition was sufficient to modulate cisplatin responsiveness. Importantly, our study extends the current understanding of lactylation in ovarian cancer by moving beyond global lactylation changes and identifying a specific non-histone lactylation event, namely ZMYM2 K529 lactylation, as a mechanistically relevant node associated with platinum resistance. Nevertheless, lactate may exert broader biological effects that are not specific to ZMYM2 lactylation. For example, lactate accumulation may influence cellular metabolism, intracellular pH, redox balance, signaling pathways, and other histone or non-histone lactylation events, all of which may contribute to treatment adaptation. Therefore, the lactate-induced phenotypes observed in this study should not be interpreted as being exclusively mediated by ZMYM2 K529 lactylation. Rather, our findings identify ZMYM2 K529 lactylation as an important mechanism through which lactate contributes to ZMYM2 stabilization and platinum-resistant phenotypes.

The present findings also suggest that interference with glycolysis–lactate metabolism may represent a feasible strategy for suppressing lactylation-dependent tumor progression and treatment resistance. In our study, glycolytic inhibition reduced the intracellular lactate levels and global protein lactylation and partially restored cisplatin sensitivity in platinum-resistant ovarian cancer cells, whereas exogenous lactate exerted the opposite effect. These results are consistent with previous studies showing that the inhibition of glycolysis can suppress lactylation and produce antitumor effects. In pancreatic ductal adenocarcinoma, glycolysis inhibitors or LDHA knockdown were shown to reduce histone lactylation and inhibit tumor growth both in vitro and in vivo [41]. In endometrial carcinoma, treatment with 2-DG or oxamate decreased histone lactylation and was accompanied by reduced proliferation and migration, increased apoptosis, and cell-cycle arrest [42]. Taken together, these observations support the notion that glycolysis inhibitors may exert antitumor and chemosensitizing effects, at least in part, through the suppression of lactate-driven lactylation events. In this context, our results further extend this concept by identifying lactylation-dependent ZMYM2 stabilization as a mechanistically relevant downstream event in platinum-resistant ovarian cancer.

A central mechanistic finding of the present study is that lactate-driven ZMYM2 K529 lactylation enhances ZMYM2 protein stability by suppressing ubiquitin–proteasome-mediated degradation. This observation is consistent with emerging evidence that lactylation can function as an active post-translational regulatory event rather than a passive metabolic mark. Recent studies have shown that lactylation may increase the stability of non-histone proteins by modulating ubiquitination-dependent turnover. Specifically, lactylation of TFEB at K91 has been reported to disrupt its interaction with the E3 ubiquitin ligase WWP2, thereby suppressing ubiquitination and proteasomal degradation [43]. In addition, lactylation of DCBLD1 has been reported to inhibit its ubiquitination and prolong its half-life [44]; and HIF-1α lactylation has been found to impair VHL-mediated recognition, thereby attenuating K48-linked ubiquitination and proteasomal degradation [45]. Notably, in the context of chemoresistance, BLM lactylation has also been reported to increase protein stability by inhibiting MIB1-mediated ubiquitination, thereby promoting DNA end resection and homologous recombination repair [46]. In the present study, lactate increased ZMYM2 protein abundance without significantly altering its mRNA expression, prolonged its half-life, and reduced its ubiquitination in a K529-dependent manner. Taken together, these findings support a model in which K529 lactylation protects ZMYM2 from proteasomal turnover and thereby sustains its elevated expression in platinum-resistant ovarian cancer. More broadly, our results provide mechanistic evidence that a site-specific lactylation event may stabilize a DNA repair-associated regulator and thereby contribute to the maintenance of a resistance-promoting cellular state.

Our functional data further indicate that ZMYM2 promotes platinum-resistant phenotypes, at least in part, by enhancing HR repair and tolerance to DNA damage. This interpretation is biologically plausible because platinum agents exert their cytotoxic effects primarily through DNA lesions, whereas HR capacity is a major determinant of cellular responsiveness to platinum-based therapy in ovarian cancer [27,29]. Previous work has shown that ZMYM2 restricts 53BP1 engagement at DNA double-strand breaks, facilitates BRCA1 loading, and thereby favors HR repair [32]. In agreement with this model, our study showed that ZMYM2 depletion reduced HR efficiency, altered post-irradiation γH2AX dynamics, increased residual γH2AX foci, and decreased tolerance to ionizing radiation and doxorubicin, whereas the restoration of ZMYM2 expression reversed these phenotypes. These findings extend the previously reported DNA repair function of ZMYM2 to the context of ovarian cancer platinum resistance and further suggest that elevated ZMYM2 not only supports cisplatin tolerance but also confers a broader adaptive advantage under DNA damage-inducing treatment conditions.

The K529R mutant further helped distinguish the lactylation-dependent regulation of ZMYM2 stability from the downstream functional activity of ZMYM2. Mutation of K529 markedly reduced ZMYM2 lactylation and attenuated lactate-induced ZMYM2 stabilization, supporting the specificity of this site in the lactate-mediated regulation of ZMYM2 protein homeostasis. However, once ZMYM2 expression was restored in rescue experiments, both the wild-type ZMYM2 and the K529R mutant were able to recover cisplatin tolerance, HR repair capacity, and DNA damage tolerance. These findings suggest that K529 lactylation primarily acts by maintaining ZMYM2 protein stability and abundance, rather than by directly determining the intrinsic repair-promoting activity of ZMYM2.

Several limitations of this study should be acknowledged. Although the present study supports an association between elevated protein lactylation and platinum-resistant phenotype as well as unfavorable clinical outcome, the potential value of protein lactylation as a biomarker for patient stratification, prognosis assessment, or therapeutic response prediction warrants further investigation in larger and independently validated cohorts. In addition, although the current data support a model in which lactate promotes ZMYM2 stabilization through K529 lactylation, the upstream regulatory machinery governing the deposition and removal of this modification remains to be elucidated. It will also be important to further define how this regulatory axis is integrated with other metabolic programs and DNA damage response networks during the development of platinum resistance. Another limitation is that concomitant metabolic conditions, such as diabetes mellitus and other disorders that may affect lactate metabolism, were not systematically incorporated into the current analysis. Future prospective studies with detailed assessment of metabolic comorbidities, systemic metabolic parameters, and direct intratumoral lactate measurements will be needed to further validate the clinical relevance of lactate-driven lactylation in ovarian cancer. Notwithstanding these considerations, the present study provides mechanistic insight into how glycolysis–lactate metabolism may be coupled to platinum resistance through the lactylation-dependent stabilization of ZMYM2. These findings therefore support the further investigation of therapeutic strategies targeting glycolysis–lactate metabolism, lactylation-dependent protein stabilization, or ZMYM2-associated DNA repair programs as potential approaches for overcoming platinum resistance in ovarian cancer.

## 4. Materials and Methods

### 4.1. Tissue Specimens and Ethical Approval

This study included clinical tissue specimens from patients with HGSOC who underwent surgery at the Department of Gynecologic Oncology, Cancer Hospital, Chinese Academy of Medical Sciences. Eligible patients were aged 18 years or older, pathologically confirmed as HGSOC, and had primary cytoreductive surgery specimens collected before the initiation of adjuvant chemotherapy. Patients were included only when complete clinicopathological information and follow-up data were available, allowing for reliable classification of platinum sensitivity or resistance according to the platinum-free interval (PFI). Formalin-fixed paraffin-embedded (FFPE) tumor tissues from 122 patients with HGSOC, including 83 platinum-sensitive and 39 platinum-resistant cases, were retrospectively collected for immunohistochemical analysis. In addition, fresh-frozen tumor tissues from 12 patients with HGSOC, including 6 platinum-sensitive and 6 platinum-resistant cases, were collected for proteomic and lactylomic analyses; among these, 10 samples (5 platinum-sensitive and 5 platinum-resistant) with sufficient remaining tissue amount were further used for Western blot analysis of global protein lactylation. All included cases were pathologically confirmed as HGSOC and were derived from primary cytoreductive surgery specimens before the initiation of adjuvant chemotherapy. Detailed clinicopathological characteristics of the FFPE cohort and the fresh-frozen HGSOC cohort are provided in Supplementary Table S1 and Supplementary Table S2, respectively. Platinum sensitivity was defined according to the platinum-free interval (PFI): recurrence within 6 months after completion of the last platinum-based chemotherapy cycle was classified as platinum-resistant disease, whereas recurrence after more than 6 months was classified as platinum-sensitive disease. This study was conducted in accordance with the Declaration of Helsinki and approved by the Ethics Committee of the National Cancer Center/National Clinical Research Center for Cancer/Cancer Hospital, Chinese Academy of Medical Sciences, and Peking Union Medical College (approval no. NCC2025C-712).

### 4.2. Cell Culture and Transfection

A2780, SKOV-3, and HEK293T cells were obtained from the Cell Resource Center, Peking Union Medical College (PCRC). A2780 and A2780-DDP cells were cultured in RPMI-1640 medium, SKOV-3 and SKOV-3-DDP cells in McCoy’s 5A medium, and HEK293T cells in high-glucose DMEM, all supplemented with 10% fetal bovine serum and 1% penicillin–streptomycin. Cells were maintained at 37 °C in a humidified incubator with 5% CO_2_. Platinum-resistant A2780-DDP and SKOV-3-DDP cells were established by stepwise exposure of parental cells to increasing concentrations of cisplatin (MCE, Monmouth Junction, NJ, USA) over 4–6 months. Resistant cells were maintained in medium containing 0.5 μg/mL cisplatin and cultured in drug-free medium for 3 days before functional assays.

Small interfering RNAs (siRNAs) targeting ZMYM2 and the corresponding negative control siRNA were synthesized by Tsingke Biotechnology (Beijing, China) and transfected using Lipofectamine RNAiMAX (Invitrogen, Carlsbad, CA, USA) according to the manufacturer’s instructions. The sequences of the siRNAs are listed in Supplementary Table S3. The expression plasmids pcDNA3-ZMYM2-Flag were used for transient overexpression experiments. Plasmids were transfected using Lipofectamine 2000 (Invitrogen, Carlsbad, CA, USA) according to the manufacturer’s instructions. pcDNA3-ZMYM2-Flag and the lentiviral vectors pLKO.1-shZMYM2-Puro and pLVX-ZMYM2-IRES-Puro were obtained from Tsingke Biotechnology (Beijing, China). The K529R mutant constructs were generated based on the corresponding wild-type vectors. Stable knockdown, overexpression, and rescue cell lines were generated by lentiviral infection followed by puromycin selection. Target cells were infected in the presence of polybrene (5 μg/mL) and selected with puromycin for 3–5 days. The sequences of the shRNAs and the main vector information are provided in Supplementary Table S3.

### 4.3. Cell Viability Assay

Cell viability was assessed using the Cell Counting Kit-8 (CCK-8; Dojindo, Kumamoto, Japan). Cells were seeded in 96-well plates at a density of 5 × 10^3^ cells per well in 100 μL complete medium and allowed to attach overnight. Cells were then treated as indicated, and blank wells for background subtraction. At the end of treatment, CCK-8 reagent was added to each well at a final volume fraction of 10%, followed by incubation for 1 h at 37 °C. Absorbance at 450 nm was measured using a microplate reader. Cell viability was normalized to the corresponding control group, and IC50 values were calculated from dose–response curves using nonlinear regression with a four-parameter logistic model in GraphPad Prism (v10.5.0).

### 4.4. Colony Formation Assay

For colony formation assays, 500–800 cells per well were seeded in 6-well plates and treated as indicated. After incubation for approximately 10–14 days, colonies were fixed and stained with crystal violet. Colonies containing more than 50 cells were counted under a light microscope. Colony numbers were normalized to the corresponding control group.

### 4.5. Cell Apoptosis Assay

Cell apoptosis was evaluated using an Annexin V-FITC/PI Apoptosis Detection Kit (Beyotime, Shanghai, China) according to the manufacturer’s instructions. Both floating and attached cells were collected, washed with PBS, and resuspended in binding buffer. A total of 1 × 10^5^ cells were stained with Annexin V-FITC and propidium iodide (PI) for 15 min at room temperature in the dark and analyzed by flow cytometry within 1 h. Data were quantified as the percentages of early and late apoptotic cells.

### 4.6. Glycolytic Activity

Glycolytic activity was assessed by measuring the ECAR using a Seahorse XF96 extracellular flux analyzer (Agilent Technologies, Santa Clara, CA, USA). Cells were seeded in Seahorse XF96 microplates at 5 × 10^3^ cells per well and incubated overnight. Before measurement, cells were washed and incubated in Seahorse XF base medium supplemented with glutamine for 1 h at 37 °C in a non-CO_2_ incubator. During the assay, glucose, oligomycin, and 2-DG were sequentially injected to final concentrations of 10 mM, 1 μM, and 20 mM, respectively. Basal glycolysis and glycolytic capacity were calculated from the ECAR curves according to the manufacturer’s instructions.

### 4.7. Intracellular Lactate Measurement

Intracellular lactate levels were measured using an L-Lactate Assay Kit (WST-8 method; Beyotime, Shanghai, China) according to the manufacturer’s instructions. Briefly, 2 × 10^6^ cells were collected, washed once with PBS, and lysed on ice in 200 μL BeyoLysis Buffer A for metabolic assay. After lysis for 10 min, samples were centrifuged at 12,000 × g for 5 min at 4 °C, and the supernatants were collected for lactate measurement. Lactate concentrations were calculated from the standard curve after the subtraction of blank values and normalized as indicated for comparative analysis.

### 4.8. Total RNA Extraction and qRT-PCR

Total RNA was extracted using an RNA extraction kit (Takara, Shiga, Japan) according to the manufacturer’s instructions. For reverse transcription, 1 μg of total RNA was converted to cDNA using the PrimeScript RT Master Mix Kit (Takara, RR036A). Quantitative real-time PCR was performed using TB Green Premix Ex Taq II (Takara, CN830A) on a QuantStudio 3 Real-Time PCR System (Thermo Fisher Scientific, Waltham, MA, USA). Relative mRNA expression was calculated using the 2^−ΔΔCt^ method with GAPDH as the internal control. Primer sequences for ZMYM2 and GAPDH are listed in Supplementary Table S4.

### 4.9. Western Blotting

Total protein was extracted from cells or tissues using RIPA lysis buffer supplemented with protease and phosphatase inhibitors. Protein concentrations were determined using a BCA protein assay kit. Equal amounts of protein were separated by SDS-PAGE and transferred onto PVDF membranes by wet transfer. Membranes were blocked with 5% non-fat milk for 1 h at room temperature and incubated overnight at 4 °C with primary antibodies against ZMYM2 (Proteintech, Wuhan, China; 24112-1-AP, 1:1000), K-lac (PTM Biolabs, PTM-1401RM, 1:1000), Flag (Proteintech, 20543-1-AP, 1:2000), γH2AX (Abcam, AB81299, 1:1000), or GAPDH (Proteintech, 60004-1-Ig, 1:5000). After washing, membranes were incubated with HRP-conjugated secondary antibodies for 1 h at room temperature. Protein bands were visualized using an enhanced chemiluminescence reagent and quantified with ImageJ (v1.54p) when indicated.

### 4.10. Immunoprecipitation (IP)

For immunoprecipitation assays, cells were lysed in pre-chilled Co-IP lysis buffer supplemented with protease and phosphatase inhibitors, and the clarified supernatants were collected as antigen samples. Protein A/G magnetic beads (MCE, Monmouth Junction, NJ, USA) were pre-washed with binding/wash buffer and incubated with the indicated antibodies to form antibody–bead complexes. After washing, the complexes were incubated with antigen sample on a rotator at room temperature for 2 h at 4 °C. The beads were subsequently washed four times with binding/wash buffer, and the bound proteins were eluted in SDS loading buffer and analyzed by Western blotting.

### 4.11. CHX Chase, Inhibitor Treatments, and Ubiquitination Assays

For CHX chase assays, cells were treated with CHX (100 μg/mL; Selleck, Houston, TX, USA) for the indicated times in the presence or absence of sodium lactate (20 mM; Sigma-Aldrich, St. Louis, MO, USA), and ZMYM2 protein levels were analyzed by Western blotting. Protein stability was evaluated by densitometric quantification of ZMYM2 relative to the corresponding loading control. For inhibitor studies, cells were treated with 2-DG (10 mM; Selleck, Houston, TX, USA) alone or in combination with MG132 (10 μM; Selleck, Houston, TX, USA) or Bafilomycin A1 (100 nM; Selleck, Houston, TX, USA), followed by Western blot analysis of ZMYM2 protein expression. For ubiquitination assays, HEK293T cells were transiently transfected with the indicated ZMYM2 constructs together with a ubiquitin expression plasmid. After treatment with sodium lactate, cell lysates were subjected to immunoprecipitation with an anti-Flag antibody, and the immunoprecipitates were analyzed by Western blotting to detect ZMYM2 lactylation and ubiquitination levels.

### 4.12. IHC

IHC staining was performed on 4-μm sections prepared from FFPE ovarian cancer tissues. After routine deparaffinization and rehydration, endogenous peroxidase activity was quenched with 3% hydrogen peroxide. Heat-induced antigen retrieval was carried out in citrate buffer (pH 6.0). The sections were then incubated overnight at 4 °C with primary antibodies against K-lac or ZMYM2 (both at 1:200 dilution). Signal detection was completed using a polymer-based HRP system, followed by DAB visualization and hematoxylin counterstaining. After dehydration, the sections were mounted for microscopic evaluation. All stained slides were reviewed independently by two investigators blinded to the clinical information. Whole-slide images were scanned and analyzed using Aperio ImageScope software, and quantitative assessment was further performed with the IHC Profiler plug-in in ImageJ [47]. For immunohistochemical assessment, both K-lac and ZMYM2 staining were quantified using the H-score system based on staining intensity and the percentage of positive tumor cells. Staining intensity was categorized into four levels: negative, weak, moderate, and strong. H-scores were calculated as follows: H-score = 1 × (% weakly positive cells) + 2 × (% moderately positive cells) + 3 × (% strongly positive cells). The resulting scores were used for subsequent association and survival analyses.

### 4.13. DR-GFP Reporter Assay

HR repair efficiency was assessed using the DR-GFP reporter system. Cells were seeded in 6-well plates and co-transfected with 500 ng of the I-SceI expression plasmid, 500 ng of the DR-GFP reporter plasmid, and 100 ng of an mCherry expression plasmid. After 48 h, cells were harvested and analyzed by flow cytometry. For gating, cell debris was first excluded based on forward scatter and side scatter profiles, and doublets were excluded using FSC-A/FSC-H gating. Successfully transfected cells were then identified as the mCherry-positive population, and GFP-positive cells were quantified within this mCherry-positive population. HR repair efficiency was expressed as the percentage of GFP-positive cells among mCherry-positive cells. Representative gating plots and GFP-positive populations are shown in Supplementary Figure S1.

### 4.14. Immunofluorescence (IF)

For IF staining, cells were seeded on sterile coverslips in 12-well plates and treated as indicated. After treatment, cells were washed with PBS, fixed with 4% paraformaldehyde for 30 min at room temperature, permeabilized with 0.1% Triton X-100 in PBS for 30 min, and blocked with 5% BSA for 30 min. Cells were then incubated with the indicated primary antibody (1:50) at 4 °C overnight, followed by incubation with the corresponding fluorescent secondary antibody for 1 h at room temperature in the dark. Nuclei were counterstained with DAPI, and coverslips were mounted with antifade mounting medium. Images were acquired using a confocal microscope under identical imaging settings. For γH2AX foci analysis, nuclear foci were quantified in 30 cells per group.

### 4.15. Proteomic and Lactylomic Analyses

Fresh-frozen tumor tissues from 12 patients with HGSOC, including 6 platinum-sensitive and 6 platinum-resistant cases, were subjected to integrated proteomic and lactylomic analyses. Tissue proteins were extracted and digested with trypsin. For lactylomic analysis, lactylated peptides were enriched using anti-lactyl lysine antibody-based affinity enrichment before LC-MS/MS in data-independent acquisition (DIA) mode. DIA proteomic data were processed using DIA-NN, and lactylomic data were analyzed using Spectronaut. Class I sites were defined as high-confidence lactylation sites with reliable localization. Differentially lactylated peptides were identified using the following criteria: fold change > 1.5 or <1/1.5 and *p* < 0.05. False discovery rate-adjusted *q* values were calculated using the Benjamini–Hochberg method to account for multiple testing. For quantitative analysis, lactylated peptide abundance was further normalized against the corresponding proteomic data to minimize the influence of changes in total protein expression and better reflect alterations in lactylation levels. Downstream analyses, including motif enrichment, hierarchical clustering, and subcellular localization analysis, were performed based on the identified differentially lactylated peptides and proteins. Candidate lactylated proteins of interest were selected according to subcellular localization, differential lactylation patterns, fold change, statistical significance, biological relevance, and feasibility of experimental validation. The processed proteomic matrix, including the quantified protein-level data used for downstream comparative analysis and interpretation, is provided as Supplementary Table S5.

### 4.16. Public Datasets and Bioinformatic Analysis

A public proteomic dataset was used to validate the differential expression of ZMYM2 protein between platinum-sensitive and platinum-resistant ovarian cancer tissues, including 14 platinum-sensitive and 12 platinum-resistant cases [33]. Survival analyses of ZMYM2 mRNA expression were performed using the Kaplan–Meier Plotter online database (ovarian cancer module) with probe 202778_s_at to evaluate PFS and OS [48].

### 4.17. Xenograft Model

Female BALB/c nude mice (6 weeks old) were used for subcutaneous xenograft assays. Cells from the indicated experimental groups were harvested in the logarithmic growth phase, resuspended in PBS/Matrigel (1:1) at a concentration of 5 × 10^7^ cells/mL, and injected subcutaneously into the right axillary region of each mouse (100 μL per mouse, 5 × 10^6^ cells). When the tumor volumes reached approximately 100 mm^3^, mice were randomly assigned to the indicated treatment groups (*n* = 4 per group) using a random number-based method and treated according to the experimental design. Cisplatin was administered intraperitoneally at 5 mg/kg every 3 days for 3 weeks. Tumor length and width were measured every 3 days by an investigator blinded to group allocation, and tumor volume was calculated as (length × width^2^)/2. At the endpoint, tumors were excised, photographed, and weighed.

All animal experiments were conducted in accordance with the institutional guidelines for the care and use of laboratory animals, and every effort was made to minimize animal suffering. The animal study protocol was approved by the Ethics Committee of the National Cancer Center/National Clinical Research Center for Cancer/Cancer Hospital, Chinese Academy of Medical Sciences, and Peking Union Medical College (approval no. NCC2025A338).

### 4.18. Statistical Analysis

Statistical analyses were performed using GraphPad Prism and SPSS (v30.0.0.0). Data are presented as mean ± standard deviation (SD) unless otherwise indicated. The number of biological replicates or analyzed samples is indicated in the corresponding figure legends. For parametric comparisons, data distribution and variance homogeneity were assessed where appropriate. Comparisons between two groups were performed using the Student’s *t*-test when the assumptions for parametric testing were met. Comparisons among multiple groups were performed using one-way ANOVA or two-way ANOVA as appropriate, followed by post hoc multiple-comparison tests when applicable. Associations between categorical variables were analyzed using the chi-square test. PFS and OS were estimated using the Kaplan–Meier method and compared by the log-rank test. A two-sided *p* value < 0.05 was considered statistically significant.

## 5. Conclusions

The present study identified a glycolysis–lactate–ZMYM2 lactylation axis as a mechanistic link between metabolic reprogramming and platinum resistance in ovarian cancer. Lactate-driven ZMYM2 K529 lactylation suppresses ubiquitin–proteasome-mediated degradation, increases ZMYM2 stability, and supports platinum-resistant phenotypes, HR repair, and tolerance to DNA damage. These findings provide new insight into the molecular basis of ovarian cancer platinum resistance and suggest that lactylation-dependent ZMYM2 stabilization may represent a potential therapeutic target.

## Data Availability

The original contributions presented in the study are included in the article/Supplementary Materials, further inquiries can be directed to the corresponding author.

## Supplementary Materials

The following supporting information can be downloaded at: https://www.mdpi.com/article/10.3390/ijms27114707/s1.

## Abbreviations

The following abbreviations are used in this manuscript:

HGSOC	High-grade serous ovarian cancer
PFS	Progression-free survival
OS	Overall survival
PFI	Platinum-free interval
FIGO	International Federation of Gynecology and Obstetrics
HR	Homologous recombination
K-lac	Protein lysine lactylation
IC50	Half-maximal inhibitory concentration
ECAR	Extracellular acidification rate
2-DG	2-deoxy-D-glucose
qRT-PCR	Quantitative real-time polymerase chain reaction
LC-MS/MS	Liquid chromatography-tandem mass spectrometry
IHC	Immunohistochemistry
IF	Immunofluorescence
IP	Immunoprecipitation
CHX	Cycloheximide
IR	Ionizing radiation
FFPE	Formalin-fixed, paraffin-embedded
SD	Standard deviation
