# Supplementary material for: Lactate-Induced ZMYM2 K529 Lactylation Stabilizes ZMYM2 and Promotes Platinum Resistance in Ovarian Cancer
Source: Int J Mol Sci. 2026 May 23;27(11):4707. doi: 10.3390/ijms27114707 (PMC13256744; doi:10.3390/ijms27114707)

Figure S1. Representative flow cytometry plots and gating strategy for the DR-GFP homologous recombination reporter assay.

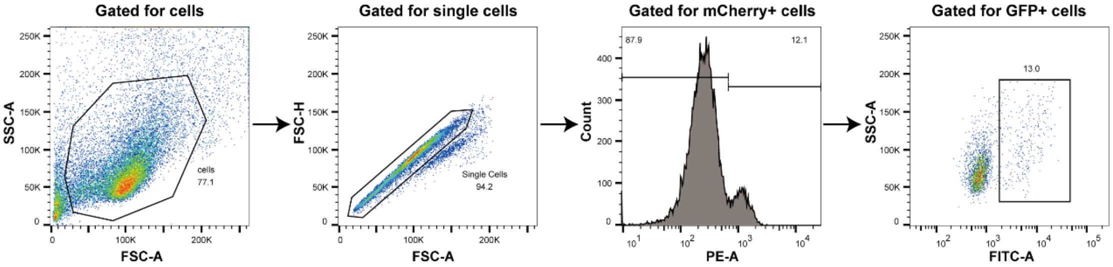

Supplement: Supplementary file 1 [file ijms-27-04707-s001.zip › Supplementary Figure S1.pdf]
