# Supplementary material for: Lactate-Induced ZMYM2 K529 Lactylation Stabilizes ZMYM2 and Promotes Platinum Resistance in Ovarian Cancer
Source: Int J Mol Sci. 2026 May 23;27(11):4707. doi: 10.3390/ijms27114707 (PMC13256744; doi:10.3390/ijms27114707)
Supplement: Supplementary file 1 [file ijms-27-04707-s001.zip › Supplementary Table S1.pdf]

Supplementary Table S1: Clinicopathological characteristics of the retrospective FFPE HGSOC cohort used for immunohistochemical analysis.

Patient ID	Age	Histology	Stage	PFI (months)	Platinum response
1	62	HGSOC	IIIC	70	Platinum-sensitive
2	61	HGSOC	IIIC	29	Platinum-sensitive
3	72	HGSOC	IIIC	15	Platinum-sensitive
4	44	HGSOC	IIIC	13	Platinum-sensitive
5	48	HGSOC	IIIC	13	Platinum-sensitive
6	50	HGSOC	IIIC	25	Platinum-sensitive
7	51	HGSOC	IIIC	28	Platinum-sensitive
8	51	HGSOC	IIIC	9	Platinum-sensitive
9	46	HGSOC	IIIC	16	Platinum-sensitive
10	72	HGSOC	IVB	77	Platinum-sensitive
11	50	HGSOC	IVA	14	Platinum-sensitive
12	64	HGSOC	IVB	9	Platinum-sensitive
13	41	HGSOC	IIIC	21	Platinum-sensitive
14	44	HGSOC	IIIC	13	Platinum-sensitive
15	54	HGSOC	IIIC	0	Platinum-resistant
16	61	HGSOC	IIIC	< 6	Platinum-resistant
17	71	HGSOC	IIIC	4	Platinum-resistant
18	53	HGSOC	IVB	< 6	Platinum-resistant
19	45	HGSOC	IVB	59	Platinum-sensitive
20	49	HGSOC	IIIC	36	Platinum-sensitive
21	51	HGSOC	IIIC	46	Platinum-sensitive
22	63	HGSOC	IIIA	60	Platinum-sensitive
23	54	HGSOC	IIIC	16	Platinum-sensitive
24	46	HGSOC	IIIC	24	Platinum-sensitive
25	52	HGSOC	IIIC	7	Platinum-sensitive
26	48	HGSOC	IVB	4	Platinum-resistant
27	58	HGSOC	IIIA	36	Platinum-sensitive
28	53	HGSOC	IIIC	< 6	Platinum-resistant
29	67	HGSOC	IIIC	3	Platinum-resistant
30	57	HGSOC	IIIC	40	Platinum-sensitive
31	33	HGSOC	IIIC	1	Platinum-resistant
32	60	HGSOC	IVB	33	Platinum-sensitive
33	67	HGSOC	IIIC	5	Platinum-resistant
34	75	HGSOC	IIIC	18	Platinum-sensitive
35	65	HGSOC	IIIC	8	Platinum-sensitive
36	68	HGSOC	IIIC	8	Platinum-sensitive
37	61	HGSOC	IIIC	4	Platinum-resistant
38	62	HGSOC	IIIC	12	Platinum-sensitive
39	50	HGSOC	IIIC	14	Platinum-sensitive
40	46	HGSOC	IIIC	2	Platinum-resistant
41	55	HGSOC	IIIC	5	Platinum-resistant

Patient ID	Age	Histology	Stage	PFI (months)	Platinum response
42	47	HGSOC	IIIC	17	Platinum-sensitive
43	58	HGSOC	IIIC	< 6	Platinum-resistant
44	54	HGSOC	IIIC	1	Platinum-resistant
45	48	HGSOC	IIIC	5	Platinum-resistant
46	60	HGSOC	IIIC	12	Platinum-sensitive
47	43	HGSOC	IIIC	5	Platinum-resistant
48	59	HGSOC	IIIB	50	Platinum-sensitive
49	51	HGSOC	IIIC	10	Platinum-sensitive
50	56	HGSOC	IIIC	8	Platinum-sensitive
51	51	HGSOC	IIIC	4	Platinum-resistant
52	59	HGSOC	IIIC	1	Platinum-resistant
53	37	HGSOC	IIIC	27	Platinum-sensitive
54	68	HGSOC	IIIC	23	Platinum-sensitive
55	66	HGSOC	IIIC	4	Platinum-resistant
56	47	HGSOC	IVB	17	Platinum-sensitive
57	51	HGSOC	IIIC	1	Platinum-resistant
58	58	HGSOC	IVB	1	Platinum-resistant
59	72	HGSOC	IIIC	17	Platinum-sensitive
60	71	HGSOC	IIIC	3	Platinum-resistant
61	60	HGSOC	IIIC	5	Platinum-resistant
62	54	HGSOC	IIIC	< 6	Platinum-resistant
63	77	HGSOC	IIIC	8	Platinum-sensitive
64	68	HGSOC	IIIC	0	Platinum-resistant
65	52	HGSOC	IIIC	25	Platinum-sensitive
66	41	HGSOC	IIIC	58	Platinum-sensitive
67	50	HGSOC	IIIC	5	Platinum-resistant
68	62	HGSOC	IIIC	11	Platinum-sensitive
69	55	HGSOC	IIIC	33	Platinum-sensitive
70	44	HGSOC	IIIC	10	Platinum-sensitive
71	44	HGSOC	IIIC	20	Platinum-sensitive
72	59	HGSOC	IIIC	10	Platinum-sensitive
73	48	HGSOC	IIIC	3	Platinum-resistant
74	47	HGSOC	IIIC	13	Platinum-sensitive
75	58	HGSOC	IIIC	9	Platinum-sensitive
76	60	HGSOC	IVA	< 6	Platinum-resistant
77	51	HGSOC	IIIC	20	Platinum-sensitive
78	70	HGSOC	IIIC	24	Platinum-sensitive
79	52	HGSOC	IIIC	8	Platinum-sensitive
80	55	HGSOC	IIIC	4	Platinum-resistant
81	43	HGSOC	IIIC	17	Platinum-sensitive
82	47	HGSOC	IIIC	40	Platinum-sensitive
83	71	HGSOC	IIIC	22	Platinum-sensitive
84	35	HGSOC	IIIC	11	Platinum-sensitive

Patient ID	Age	Histology	Stage	PFI (months)	Platinum response
85	58	HGSOC	IIIC	16	Platinum-sensitive
86	46	HGSOC	IIIB	51	Platinum-sensitive
87	36	HGSOC	IIIC	9	Platinum-sensitive
88	71	HGSOC	IIIC	38	Platinum-sensitive
89	68	HGSOC	IIIC	4	Platinum-resistant
90	48	HGSOC	IIIB	96	Platinum-sensitive
91	40	HGSOC	IIIC	10	Platinum-sensitive
92	61	HGSOC	IIIA	4	Platinum-resistant
93	53	HGSOC	IIIC	29	Platinum-sensitive
94	43	HGSOC	IIIC	24	Platinum-sensitive
95	60	HGSOC	IIIC	29	Platinum-sensitive
96	41	HGSOC	IIIC	25	Platinum-sensitive
97	57	HGSOC	IIIC	9	Platinum-sensitive
98	59	HGSOC	IVB	9	Platinum-sensitive
99	59	HGSOC	IIIC	< 6	Platinum-resistant
100	51	HGSOC	IIIB	9	Platinum-sensitive
101	46	HGSOC	IIIC	8	Platinum-sensitive
102	57	HGSOC	IIIC	15	Platinum-sensitive
103	60	HGSOC	IIIC	1	Platinum-resistant
104	71	HGSOC	IIIC	10	Platinum-sensitive
105	51	HGSOC	IVB	4	Platinum-resistant
106	54	HGSOC	IIIC	19	Platinum-sensitive
107	43	HGSOC	IIIC	26	Platinum-sensitive
108	61	HGSOC	IIIC	10	Platinum-sensitive
109	46	HGSOC	IIIC	12	Platinum-sensitive
110	48	HGSOC	IIIB	13	Platinum-sensitive
111	65	HGSOC	IIIC	< 6	Platinum-resistant
112	41	HGSOC	IIIC	37	Platinum-sensitive
113	57	HGSOC	IIIC	15	Platinum-sensitive
114	46	HGSOC	IIIC	13	Platinum-sensitive
115	54	HGSOC	IIIC	42	Platinum-sensitive
116	55	HGSOC	IVB	4	Platinum-resistant
117	71	HGSOC	IIIC	4	Platinum-resistant
118	64	HGSOC	IIIB	17	Platinum-sensitive
119	46	HGSOC	IIIC	62	Platinum-sensitive
120	59	HGSOC	IVB	< 6	Platinum-resistant
121	63	HGSOC	IIIC	8	Platinum-sensitive
122	64	HGSOC	IVB	0	Platinum-resistant
