# Supplementary material for: Lactate-Induced ZMYM2 K529 Lactylation Stabilizes ZMYM2 and Promotes Platinum Resistance in Ovarian Cancer
Source: Int J Mol Sci. 2026 May 23;27(11):4707. doi: 10.3390/ijms27114707 (PMC13256744; doi:10.3390/ijms27114707)
Supplement: Supplementary file 1 [file ijms-27-04707-s001.zip › Supplementary Table S2.pdf]

Supplementary Table S2: Clinicopathological characteristics of the fresh-frozen HGSOC cohort used for proteomic and lactylomic analyses

Sample ID	Age	Histology	FIGO stage	PFI (months)	Tissue use
SS1	62	HGSOC	IIIC	> 36	Western blot, Proteomics and Lactylomics
SS2	74	HGSOC	IIIB	> 27	Western blot, Proteomics and Lactylomics
SS3	59	HGSOC	IIIC	> 39	Western blot, Proteomics and Lactylomics
SS4	55	HGSOC	IIIA	> 20	Western blot, Proteomics and Lactylomics
SS5	63	HGSOC	IIIC	> 31	Western blot, Proteomics and Lactylomics
SS6	58	HGSOC	IVB	44	Proteomics and Lactylomics
SR1	63	HGSOC	IIIC	5	Western blot, Proteomics and Lactylomics
SR2	57	HGSOC	IIIC	4	Western blot, Proteomics and Lactylomics
SR3	65	HGSOC	IIIB	4	Western blot, Proteomics and Lactylomics
SR4	64	HGSOC	IIIB	5	Western blot, Proteomics and Lactylomics
SR5	68	HGSOC	IIIC	2	Western blot, Proteomics and Lactylomics
SR6	70	HGSOC	IVA	2	Proteomics and Lactylomics
