# Supplementary material for: Lactate-Induced ZMYM2 K529 Lactylation Stabilizes ZMYM2 and Promotes Platinum Resistance in Ovarian Cancer
Source: Int J Mol Sci. 2026 May 23;27(11):4707. doi: 10.3390/ijms27114707 (PMC13256744; doi:10.3390/ijms27114707)
Supplement: Supplementary file 1 [file ijms-27-04707-s001.zip › Supplementary Table S3.pdf]

Supplementary Table S3: Sequences of siRNAs and shRNA targeting ZMYM2.

Name	Sequence （5'-3'）
Non-silencing	UUCUCCGAACGUGUCACGUTT
ZMYM2 siRNA1	GCAAGGUGUGACUGUUGUAAA
ZMYM2 siRNA2	CCAGAAUGCUCUAGUUCUACA
ZMYM2 siRNA3	GGAGCUCUAAAUAAAUCAAGA
ZMYM2 shRNA	GCAAGGTGTGACTGTTGTAAA
