# Supplementary material for: Lactate-Induced ZMYM2 K529 Lactylation Stabilizes ZMYM2 and Promotes Platinum Resistance in Ovarian Cancer
Source: Int J Mol Sci. 2026 May 23;27(11):4707. doi: 10.3390/ijms27114707 (PMC13256744; doi:10.3390/ijms27114707)
Supplement: Supplementary file 1 [file ijms-27-04707-s001.zip › Supplementary Table S4.pdf]

Supplementary Table S4: Primer sequences used for qRT-PCR.

qRT-PCR Primer	Sequence (5'-3')
Human GAPDH Forward Sequence	GTCTCCTCTGACTTCAACAGCG
Human GAPDH Reverse Sequence	ACCACCCTGTTGCTGTAGCCAA
Human ZMYM2 Forward Sequence	TGTTCCAGTGCCTATCCCTGTG
Human ZMYM2 Reverse Sequence	TCTCACTGCTGTCCAATGGAGC
